# Physiological Responses Induced by Manual Therapy in Animal Models: A Scoping Review

**DOI:** 10.3389/fnins.2020.00430

**Published:** 2020-05-08

**Authors:** Carla Rigo Lima, Daniel Fernandes Martins, William Ray Reed

**Affiliations:** ^1^Rehabilitation Science Program, University of Alabama at Birmingham, Birmingham, AL, United States; ^2^Postgraduate Program in Health Sciences, Experimental Neuroscience Laboratory (LaNEx), University of Southern Santa Catarina, Palhoça, Brazil; ^3^Department of Physical Therapy, University of Alabama at Birmingham, Birmingham, AL, United States

**Keywords:** joint mobilization, massage, spinal manipulation, manual therapy, physical medicine, animals, pain, scoping review

## Abstract

**Background:** Physiological responses related to manual therapy (MT) treatment have been investigated over decades using various animal models. However, these studies have not been compiled and their collective findings appraised. The purpose of this scoping review was to assess current scientific knowledge on the physiological responses related to MT and/or simulated MT procedures in animal models so as to act as a resource to better inform future mechanistic and clinical research incorporating these therapeutic interventions.

**Methods:** PubMed, Cumulative Index to Nursing and Allied Health Literature (CINAHL), Cochrane, Embase, and Index of Chiropractic Literature (ICL) were searched from database inception to August 2019. Eligible studies were: (a) published in English; (b) non-cadaveric animal-based; (c) original data studies; (d) included a form of MT or simulated MT as treatment; (e) included quantification of at least one delivery parameter of MT treatment; (f) quantification of at least one physiological measure that could potentially contribute to therapeutic mechanisms of action of the MT. MT studies were categorized according to three main intervention types: (1) mobilization; (2) manipulation; and (3) massage. Two-phase screening procedures were conducted by a pair of independent reviewers, data were extracted from eligible studies and qualitatively reported.

**Results:** The literature search resulted in 231 articles of which 78 met inclusion criteria and were sorted by intervention type. Joint mobilization induced changes in nociceptive response and inflammatory profile, gene expression, receptor activation, neurotransmitter release and enzymatic activity. Spinal manipulation produced changes in muscle spindle response, nocifensive reflex response and neuronal activity, electromyography, and immunologic response. Physiological changes associated with massage therapy included autonomic, circulatory, lymphatic and immunologic functions, visceral response, gene expression, neuroanatomy, function and pathology, and cellular response to *in vitro* simulated massage.

**Conclusion:** Pre-clinical research supports an association between MT physiological response and multiple potential short-term MT therapeutic mechanisms. Optimization of MT delivery and/or treatment efficacy will require additional preclinical investigation in which MT delivery parameters are controlled and reported using pathological and/or chronic pain models that mimic neuromusculoskeletal conditions for which MT has demonstrated clinical benefit.

## Introduction

Manual therapy (MT) techniques are considered one of the oldest interventions in medicine (Lennard et al., 2011) and may be defined as passive movements or forces applied to joints and soft tissues, often delivered by hand (American Physical Therapy Association, 1999; American Academy of Ortopaedic Manual Physical Therapists, 2008). Examples of MT techniques include, but are not limited to, mobilization, manipulation, and massage exhibiting both demonstrated and purported physiological and/or psychological benefits including increased range of motion and tissue extensibility, reduction of pain, inflammation and swelling, and/or relaxation (American Physical Therapy Association, 1999; American Academy of Ortopaedic Manual Physical Therapists, 2008). Initially, mechanistic investigations regarding the effects of MT were primarily biomechanically focused however, a recent shift toward neurophysiological and psychological effects of MT has been observed in the literature (Bialosky et al., 2009, 2018; Lennard et al., 2011; Vigotsky and Bruhns, 2015).

Clinical benefits of MT has been reported for a wide variety of conditions including hip (MacDonald et al., 2006) and knee osteoarthritis (Deyle et al., 2000), low back pain (Licciardone et al., 2003; Childs et al., 2004; Skelly et al., 2018), carpal tunnel syndrome (Rozmaryn et al., 1998; Akalin et al., 2002), among others. Despite therapeutic benefits and high patient satisfaction (Seferlis et al., 1998; Burke et al., 2007) observed with MT treatments, appropriate utilization, and/or patient referral of these interventions by healthcare providers remains low (Li and Bombardier, 2001; Bishop and Wing, 2003). In an attempt to address this issue, clinical prediction rules for identifying individuals likely to benefit from MT have been proposed but many have not been validated and thus need to be interpreted with caution (Flynn et al., 2002; Childs et al., 2004; Cleland et al., 2006, 2007; Vicenzino et al., 2009; Puentedura et al., 2012). Although the development of prediction rules based on signs and symptoms might prove beneficial to clinical practice, a greater understanding of underlying MT physiological responses and mechanisms of action will most likely be required in order to identify those individuals that will respond better to MT interventions.

Animal studies are conducted in a variety of health-related areas, such as drug and biomedical research (Hooijmans et al., 2018), with the intent to provide greater knowledge regarding physiological mechanisms, biological effects, and dose-response relationships of particular therapeutic interventions (Hackam and Redelmeier, 2006). Despite certain translational limitations, animal models continue to be acknowledged as essential to advancing scientific knowledge and mechanistic understanding of pharmacological and non-pharmacological therapeutic interventions (Jucker, 2010; Kitta et al., 2018). A comprehensive review of physiological responses associated with MT will better inform future research efforts within the field that may ultimately lead to increased MT therapeutic efficacy and appropriate utilization by healthcare providers.

## Materials and Methods

A scoping review methodology was selected in order to compile and appraise data pertaining to our research question and to offer new insights by comprehensively examining the current state of scientific knowledge available in the literature while identifying gaps which need to be addressed. The chosen framework was based on scoping review guidelines by Arksey and O'Malley (2005) and Preferred Reporting Items for Systematic reviews and Meta-Analyses extension for scoping reviews (PRISMA-ScR Supplementary Data Sheet 1; Tricco et al., 2018).

### Step 1: Identifying the Research Question

The purpose of this scoping review was to identify physiological responses related to MT, and/or simulated MT, that have been investigated using animal models.

### Step 2: Identifying Relevant Studies

**Figure 1:**
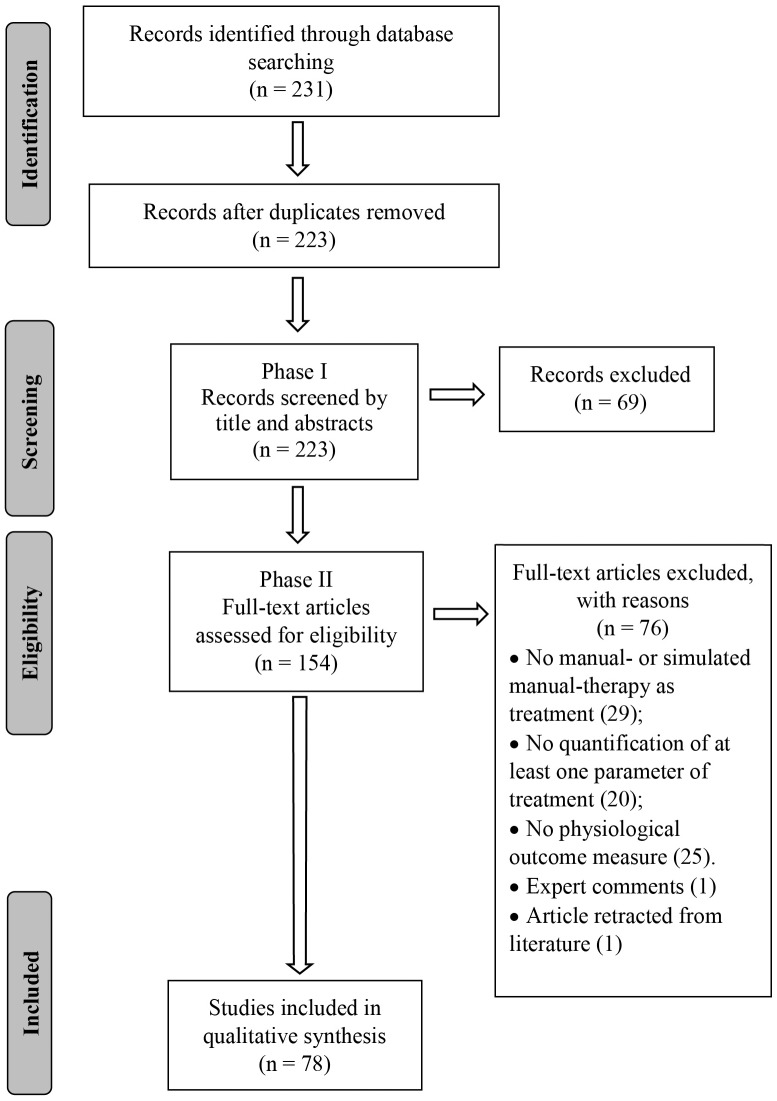
Flowchart diagram.

A search strategy was developed with the assistance of a research librarian and the following databases were searched from their inception to August 2019: PubMed, Cumulative Index to Nursing and Allied Health Literature (CINAHL), Cochrane, Embase and Index of Chiropractic Literature (ICL). A combination of keywords and indexing terms relevant to three topics (physiological responses, MT inventions and animal models) was first created for PubMed and subsequently adapted to the other databases (Supplementary Data Sheet 2). An EndNote (version X9.2, Clarivate Analytics, Boston, MA, USA) library was created, duplicates excluded and the PRISMA flow chart used to report the number of selected/excluded studies throughout the review process (Figure 1).

### Step 3: Study Selection

#### Inclusion and Exclusion Criteria

Inclusion criteria consisted of: (a) studies published in English; (b) non-cadaveric animal-based studies; (c) original data studies; (d) included a form of MT or simulated MT as a treatment/intervention; (e) included quantification of at least one delivery parameter of MT treatment (i.e., force, amplitude, direction, duration, or frequency); (f) outcomes included at least one physiological measure that potentially could contribute to therapeutic mechanisms of action of MT. Studies were excluded if classified as: human/clinical studies, practice guidelines, unpublished manuscripts, dissertations, reviews, expert comments, book and/or book chapters, government reports, conference proceedings, reported only nociceptive behavior, or biomechanical-related data.

#### Screening and Agreement

Search results were screened in two phases. Phase I consisted of title and abstract screening in order to include possible relevant studies and exclude irrelevant ones. Phase II consisted of full text screening of studies previously identified as possibly relevant in order to select eligible studies. Screenings, in both Phase I and II, were conducted by two independent reviewers (CRL, WRR) and any discrepancy regarding study eligibility was mediated by a third reviewer (DFM, *n* = 4).

### Step 4: Data Charting

The following data were extracted from eligible studies: author(s), year of publication, purpose of the study, keywords, language, animal species, type of MT or simulated MT implemented, intervention parameters, and main physiological outcomes reported. Extraction parameters were jointly defined a priori by two authors (CRL, WRR). Data extraction was then performed by one author (CRL), and verified by a second author (WRR) to account for error minimization.

### Step 5: Collating, Summarizing, and Reporting the Results

Data were descriptively summarized according to the following data items:

1. **Basic numerical analysis**: number of studies and trends regarding year of publication.
2. **Summary of findings by intervention type:** average ratio of delivery parameters reported, species selected, and physiological responses associated to each MT or simulated MT being used.
3. **Implication of the results:** we reported review findings according to three common MT intervention types (mobilization, manipulation, and massage therapy) identified in our search in order to facilitate experimental design and translational implications for future investigations.

For the purpose of this review, the operational definitions listed in Table 1 were considered when summarizing and reporting the results.

**Table 1:** Operational definitions.

**Variable**	**Operational Definition**
Mobilization techniques	Defined as slow repetitive non-thrust oscillatory motions of varying amplitudes (Xia et al., 2016).
Manipulation therapy	Defined as a single high-velocity low-amplitude thrust targeting a joint (Xia et al., 2016).
Massage therapy	Defined as the mechanical stimulation of soft tissues (Moyer et al., 2004).
Force	Defined as magnitude of strength or energy applied to a tissue/joint.
Amplitude	Defined as the range or depth of the movement being applied to a tissue/joint.
Direction	Defined as the direction of the movement being performed.
Duration	Defined as the length of treatment.
Movement frequency	Defined as the rate of movement being applied to a tissue/joint.

## Results

### Basic Numerical Analysis

**Figure 2:**
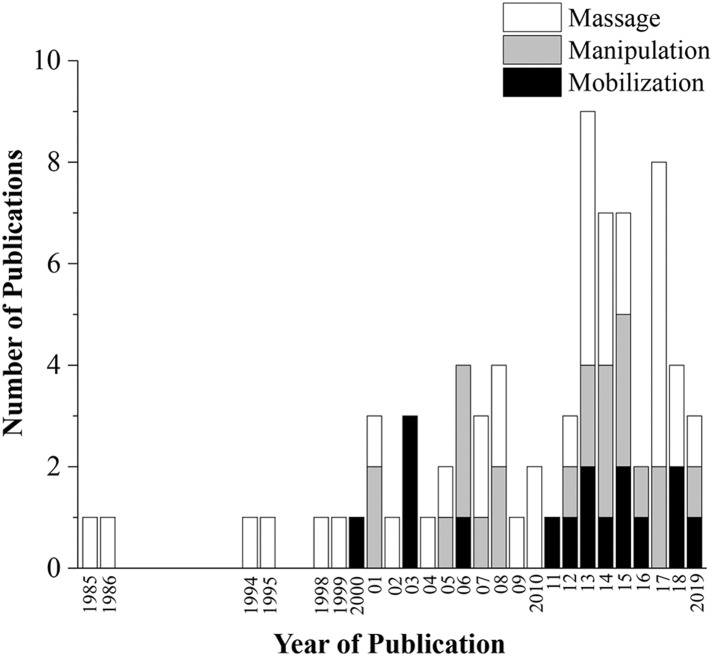
Number of publications per year. Hybrid studies not included.

The database search conducted on August 16th, 2019 resulted in 231 articles. After duplicates were removed, 223 articles had their titles and abstracts screened on Phase I and 154 articles were considered relevant for eligibility screening on Phase II. Of these 154 articles, 76 were excluded because of failure to meet all eligibility requirements leaving a total of 78 articles included in this review (Figure 1). Seventeen articles (21.8%) were classified as mobilization studies, 21 (26.9%) as manipulation studies, 37 (47.4%) as massage studies, and 3 (3.8%) as hybrid studies where the physiological outcomes were interpreted in conjunction with another intervention (e.g., exercise + MT). More than half (53%) of the articles were published in the last 7 years (Figure 2).

### Mobilization

A total of 17 studies investigated underlying physiological effects associated with mobilization interventions. The number of studies reporting a specific MT delivery parameter (i.e., force, amplitude, direction, duration, and movement frequency) relative to the total number of articles reviewed is shown in Table 2. Species selected for mobilization interventions included: cats (1/17), mice (3/17), rabbits (4/17), and rats (9/17). Anatomical site of mobilization included the intervertebral joint (1/17), knee joint (7/17), ankle joint (8/17), and one multi-joint intervention (squatting and standing movements). Detailed information regarding the mobilization delivery parameters, species and outcome measures utilized can be found in Supplementary Data Sheet 3.

**Table 2:** Ratio of parameters reported.

	**Force**	**Amplitude**	**Direction**	**Duration**	**Movement Frequency**
Mobilization	2/17	15/17	17/17	16/17	6/17
Manipulation	19/21	2/21	19/21	18/21	2/21
Massage	16/37	2/37	26/37	37/37	16/37
Hybrid	0/3	1/3	3/3	3/3	0/3

#### Ankle Joint Mobilization

A total of eight studies investigated the effects of ankle joint mobilization (AJM) on physiological outcomes (Martins et al., 2011, 2012, 2013a,b; da Silva et al., 2015; Santos et al., 2018; Zhu et al., 2018; Salgado et al., 2019). Of these eight studies, four investigated the effects of AJM on measures related to neuropathic pain (Martins et al., 2011; da Silva et al., 2015; Santos et al., 2018; Zhu et al., 2018). Collectively, these studies reported that AJM resulted in the following: (a) reduced glial markers' expression (monoclonal mouse anti-rat CD11b/c and glial fibrillary acidic protein—GFAP) and increased myelin sheath thickness in the sciatic nerve (Martins et al., 2011), (b) significant improvement in sciatic nerve regeneration following injury mediated by nerve growth factor (NGF) and myelin protein zero (MPZ) (da Silva et al., 2015), (c) significant reduction of substance P and transient receptor potential vanilloid 1 (TRPV1) levels in the dorsal root ganglions (DRGs) (Santos et al., 2018), and (d) significant increase in μ-opioid receptor (Santos et al., 2018) and significant difference in interleukin (IL)-1β level in the nerve trunk and branches limited to treated vs. non-treated sides (Zhu et al., 2018).

Four studies investigated the effects of AJM in post-operative models (Martins et al., 2012, 2013a,b; Jielile et al., 2016). Martins et al. (2012) assessed the effects of AJM on opioid receptor and leukocyte migration after naloxone and fucoidan injections, respectively in a post-operative pain model. The reduction in mechanical nocifensive reflex observed with 9 min of daily AJM, but not three, was mediated by opioid receptor availability. Martins et al. (2013a) investigated the contribution of cannabinoid receptors to the AJM anti-hyperalgesic effects. Central blockage of cannabinoid receptor 1 ended, and peripheral blockage of cannabinoid receptor 2 reversed the analgesic effects observed with AJM, thereby suggesting endocannabinoid system involvement in AJM-induced anti-hyperalgesia. In addition, the adenosinergic system was also found to play a role in the anti-hyperalgesic effects of AJM with these effects being mediated by adenosinergic receptors, adenosine A1 and α-2-adrenergic receptors and serotonergic pathways (Martins et al., 2013b). The last study assessed the effects of AJM on oxidative stress, mitochondrial function, protein carbonyls, antioxidant enzymes superoxide dismutase (SOD), and catalase (CAT) levels in a chronic post-ischemia pain model of complex regional pain syndrome type I. AJM-related reduction in pain behavior was attributed to the prevention of oxidative stress (malondialdehyde) and an increase in protein carbonyls and CAT (Salgado et al., 2019). Jielile et al. (2016), assessed the protein expression involved in Achilles tendon healing observed with early AJM in a post-operative model. Collapsin response mediator protein 2 (CRMP-2), galactokinase 1, tropomyosin-4, and transthyretin were identified as potential proteins involved in the tissue healing process observed with early mobilization.

#### Knee Joint Mobilization

A total of seven studies investigated the effects of knee joint mobilization (KJM) on physiological outcomes (Ip et al., 2000; Malisza et al., 2003a,b; Skyba et al., 2003; Ferretti et al., 2006; Ruhlen et al., 2014; Wang et al., 2015). Three studies examined gene transcriptional expression in knee inflammatory models (Ferretti et al., 2006; Ruhlen et al., 2014; Wang et al., 2015). Ferretti et al. (2006) looked at physiological responses (gene transcriptional activation and IL-10 expression) of joint mobilization in a knee osteoarthritis model of inflammation. Joint mobilization inhibited the transcriptional activation of pro-inflammatory genes [IL-1β, cyclooxygenase (COX)-2, and matrix metalloproteinase (MMP)-1] and upregulated IL-10 expression consequently improving local inflammation. Additionally, Wang et al. (2015) reported that a 9% elongation of the sciatic nerve by performing knee flexion/extension (30°-130°), 10 times daily for a total of 4 weeks, significantly reducing muscle ring finger (MuRf)-1 expression and slowing muscle atrophy. Conversely, Ruhlen et al. (2014) found no statistically significant differences in gene expression induced by KJM in the rat's spinal cord.

Skyba et al. (2003) investigated the involvement of neurotransmitter's receptors (γ-aminobutyric acid—GABA_A_; opioid; α_2_-adrenergic; and 5-HT_1/2_ receptors) on mechanical withdrawal threshold observed with KJM (3 × 3 min; 1 min rest) in a capsaicin-induced pain model. Blockade of 5-HT_1/2_ and α_2_-adrenergic receptors, respectively, prevented and reversed the anti-hyperalgesic effect of KJM. Additionally, the blockade of GABA_A_ and opioid receptors did not significantly affect the anti-hyperalgesia observed with KJM.

Two studies (Malisza et al., 2003a,b) used functional magnetic resonance imaging (fMRI) to assess the analgesic effects of KJM (3 × 3 min; 1 min rest) at the cerebral and spinal cord level. Injection of capsaicin into the ankle joint or into the plantar surface of the hind-paw bilaterally activated pain processing areas within the brain (anterior cingulate, frontal cortex and sensory motor cortex) and spinal cord dorsal horn. KJM, however, did not significantly affect the brain or spinal cord activations in comparison to control animals receiving no mobilization. Additionally, Kang et al. (2001) found that slow spinal (L6) ramp and hold loading (at 25%, 50%, 75%, and 100% body weight) following chemosensitive afferent stimulation (subfascial or intramuscular injection of bradykinin/capsaicin) failed to alter paraspinal muscle spindle sensitivity.

### Manipulation

A total of 21 studies investigated physiological responses for manipulation-related interventions. The number of studies reporting the specific MT delivery parameters (i.e., force, amplitude, direction, duration, and movement frequency) relative to the total number of articles reviewed is shown on Table 2. Species chosen for manipulative interventions included: dogs (1/21), sheep (3/21), rats (6/21), and cats (11/21). Site of intervention was unanimously the spine. Detailed information regarding the manipulation parameters, species and outcome measures being utilized can be found in Supplementary Data Sheet 3. Out of the 21 studies, 18 investigated electrophysiological aspects of spinal manipulation (SM) on physiological outcomes (Pickar and Wheeler, 2001; Sung et al., 2005; Pickar and Kang, 2006; Song et al., 2006, 2016; Pickar et al., 2007; Colloca et al., 2008; Cao et al., 2013; Reed et al., 2013, 2014a,b,c, 2015a,b, 2017a,b; Reed and Pickar, 2015; Duarte et al., 2019).

#### Muscle Spindle Afferent Response

Investigating paraspinal (multifidus and longissimus) muscle spindle response during the SM thrust, Pickar et al. found that lumbar SM (Posterior-to-Anterior direction—PA; 0, 200, 400, and 800 ms; 33, 66, or 100% body weight—BW thrust magnitude) demonstrated an abrupt increase in spindle discharge as thrust duration approached 100 ms which has clinical relevancy to manually delivered SM (Sung et al., 2005; Pickar and Kang, 2006; Pickar et al., 2007). They also noted higher spindle sensitivity to 1 mm vertebra displacements compared to 2 mm displacements during SM (Pickar et al., 2007). Additional studies by this group (Cao et al., 2013; Reed et al., 2013, 2014a,b,c, 2015a,b, 2017a,b; Reed and Pickar, 2015) extensively investigated paraspinal muscle spindle response to varied combinations of manipulative thrust durations, thrust magnitudes, thrust directions, anatomical location, as well as the impact of soft tissue preload and lumbar facet joint fixation on spindle response.

Reed et al. (2014a) investigated how soft tissue preload (18 and 43% of peak applied thrust force; at 1 or 4 s duration) prior to SM delivery affected lumbar spindle responses during and after SM (55% BW; 75 ms thrust duration). Smaller preload magnitudes and longer preload durations significantly increased spindle discharge during the manipulative thrust. The highest preload magnitude and longest duration led to a significantly greater mean decrease in resting spindle discharge following SM, but these decreases were fairly modest in magnitude.

Reed and Pickar (2015) investigated the effects of 4 specific anatomic SM thrust locations (L6 spinous process, L6 lamina, L6 inferior articular process, and L7 spinous process) on L6 spindle response. L6 SM (peak force of 21.3N; 100 ms pulse duration; PA direction) significantly increased spindle discharge at all L6 contact sites compared to the L7 contact site. However, there were no statistically significant differences between spindle response during SM between any of the L6 SM contact sites (Reed et al., 2015b). In addition, L6 spindle afferent response to SM thrust duration (55% BW; at 0, 75, 100, 150, and 250 ms) delivered at either L4 or L6 in different spinal joint conditions (L6 lumbar laminectomy-only followed by single and/or multiple spinal levels of unilateral facet fixation) was investigated. Independent of spinal joint condition, shorter L6 thrust durations (≤150 ms) elicited the greatest change in mean L6 spindle response. They also found that L4 SM elicited 60–80% of the L6 spindle response compared to when SM was delivered at the L6 spinous process in the presence or absence of spinal facet joint fixation. Together, these findings demonstrated for the first time the existence of a regional mechanoreceptor response gradient related to SM delivery and a proportional decrease in muscle spindle response to SM directly related to increases in spinal joint quasi-stiffness.

Muscle spindle response to extremely short (2–3 ms) SM thrust durations using two commercially available SM devices (Activator® and Pulstar®) were also investigated (Reed and Pickar, 2015; Reed et al., 2017b). Preliminary data suggests that post-SM thrust spindle responses are thrust parameter and device specific. These extremely short thrust durations decreased muscle spindle discharge upon delivery and depending on device force setting, between 44 and 80% (Pulstar®) and 11–63% (Activator®) of spindle responses required prolonged periods (>6 s) to return to within 95% of baseline mean frequency discharge (Reed et al., 2017b).

#### Neuronal Activity

Reed and colleagues also investigated the effects of different SM thrust magnitudes (0, 55, and 85% BW; at 100 ms pulse duration) and thrust durations (100 and 400 ms; at 85% BW) on mechanical trunk thresholds (PA; 45° caudal-ward; 45° cranial-ward directions) in lateral thalamic neurons (wide dynamic range—WDR; nociceptive specific—NS) (Reed et al., 2014b,c). There was a significant increase in mechanical trunk threshold among NS neurons at 85% BW in comparison to 0% (non-thrust) in the PA direction while no statistically significant differences were found among WDR neurons (Reed et al., 2014b). Additionally, SM thrust duration appeared not to impact the mechanical trunk threshold of NS lateral thalamic neurons (Reed et al., 2014c). Reed et al. (2017a), investigated the effect of lumbar SM (85% BW; 100 ms pulse duration; in PA direction) on spontaneous and noxiously evoked activity in medial thalamic submedius neurons. A significant reduction in spontaneous activity was found to occur 180–240 s following an L5 lumbar thrust and inhibitory evoked responses in the contralateral hind-paw were attenuated compared to non-thrust controls.

Song et al. (2006) demonstrated changes in mechanical and thermal sensitivity with SM (<0.1 ms; daily for 7 days and every other day for the following week) following lumbar inflammation that was induced by the injection of an inflammatory soup (bradykinin, 5-HT, histamine, and prostaglandin) into the L5 intervertebral foramen. *In vitro* electrophysiological recordings from L5 DRG neurons from these animals were performed and SM resulted in a significant reduction in neuronal hyperexcitability following induced tissue inflammation.

#### Electromyography

Colloca et al. (2006) investigated effects of varied force-time profiles of SM on multifidus muscle electromyographic (EMG) response in adolescent sheep. A significant increase in EMG response was recorded when the manipulative force was increased (20, 40, 60N) and SM thrust duration held constant (100 ms). On the other hand, no statistically significant differences were found for different thrust durations (10, 100, 200 ms) at constant force (80N). Similar EMG responses to SM were also found in animals with healthy or degenerative discs (Colloca et al., 2008). EMG responses (multifidus muscle) to SM (80N at 10 or 100 ms) in different models of spinal lesions (spondylolytic defects and annular lesions) were also determined (Colloca et al., 2012). Significant differences in EMG positive response were found between the annular lesion's group and its control (with the degenerative model expressing 25–30% reduction in positive EMG response), while no statistically significant EMG differences were found between the spondylosis group and its control (Colloca et al., 2012).

#### Immunologic Response

Song et al. (2016) determined the effects of SM (<0.1 ms pulse duration; rostrally at 40°-50° to the vertebral horizontal line) on a neuroinflammatory profile [neuron excitability, c-Fos and protein kinase C (PKC)-γ expression, IL-1β, TNF-α, IL-10] at the DRG and spinal cord level in both a neuropathic and post-operative pain models. SM significantly reduced DRG neuron hyperexcitability, c-Fos and PKCγ expression, as well as IL-1β in DRG neurons. Additionally, a significant increase in IL-10 levels was observed in the spinal cord. Just recently, Duarte and colleagues showed that SM prevented increases in lipid hyperoxides as well as nitric oxide (NO) metabolites and reduced CAT enzymatic activity in a knee immobilization pain model (Duarte et al., 2019).

In addition to the above studies, Wynd et al. (2008) reported that a series of 20 cervical manipulations failed to alter the area, length, or volume of a pre-existing experimentally-induced canine vertebral artery lesion.

### Massage

A total of 37 studies investigated physiological responses to various massage interventions. The number of studies reporting specific MT parameters (i.e., force, amplitude, direction, duration, and movement frequency) relative to the total number of articles reviewed is shown in Table 2. Species chosen for massage interventions included: sheep (1/27), cats (1/27), mice (5/27), rabbits (6/27), and rats (24/27). Anatomic sites of intervention included various parts of the body (i.e., back, neck, eyelids, abdomen, and upper limb). Detailed information regarding the massage delivery parameters, species and outcome measures utilized can be found in Supplementary Data Sheet 3.

#### Autonomic and Circulatory-Related Responses

Kurosawa et al. (1995) looked at the effects of abdominal massage (ventral and/or lateral regions, 20 cm/s, 0.017–0.67 Hz, 100–150 mmH2O, for either 1 or 5 min) on arterial blood pressure (carotid artery) in anesthetized rats. Stroking the ventral or both the ventral and lateral abdominal regions for 1 min significantly reduced arterial blood pressure (50 mmHg) to a greater degree than by stroking the lateral side alone (30 mmHg) with blood pressure returning to baseline levels within 1 min after massage cessation. Lund et al. (1999) determined the effects of massage on blood pressure and heart rate (HR) in unanesthetized rats. Massage-like stroking maneuvers were delivered for either 2 or 5 min to the rat's abdomen or back. Five minutes of abdominal massage reduced blood pressure by 20mmHg and HR by 60 beats/min for up to 4 h, while 2 min of massage produced less pronounced effects. Spurgin et al. (2017) used an adapted sphygmomanometer to deliver controlled abdominal massage (20 or 40 mmHg; 5 min) to two strains of rats. Systolic blood pressure was significantly reduced at various time intervals following 20 and 40 mmHg massage in both strains with 40 mmHg yielding a greater reduction in both strains.

#### Lymphatic and Immune Response

Wolf et al. (1994) investigated the lymphotropic effects of massage by following the transport of nanoparticles using quantitative lymphography. After injection of labeled nanoparticles, the hind-paw was massaged for 9 min and quantitative lymphography of popliteal, presacral, and paraaortic nodes conducted every 10 min. Massaging the injection site significantly increased lymph flow rate and lymph node nanoparticles' accumulation demonstrating that gentle massage proved to be a powerful lymphotropic stimulus. Trubetskoy et al. (1998) studied the effects of massage (5 min) on liposome transportation via lymphatic pathways. Liposomes (200 nm) with incorporated angiotensin II (polyethyleneglycol-distearoyl phosphatidyl ethanolamine—PEG-PE; Plain egg phosphatidyl choline—EPC; and non-encapsulated angiotensin II) were injected in the front paw of New Zealand white rabbits and planar gamma-images of the rabbits' upper body were taken at 20 and 25 min after injection. Massage of the injection site was delivered between the two gamma recordings and significantly increased the percentage (40%) of injected liposome dose in the blood indicating that liposome release into the bloodstream is an event triggered by massage.

Major et al. (2015) investigated the immunologic modulation effects of massage (brush or hand stroking from thoracic to superior hind-limb region; 60 min for a total of 8 days) in mice. Significant increases in thymocyte number as well as CD4^+^CD8^+^, CD4^+^, and CD8^+^ subpopulations for the hand-massaged group were found in comparison to control (no massage/handling). These changes were accompanied by a significant reduction in noradrenergic innervation of lymphoid organs. No statistically significant differences were observed for the brush-massage group. Waters-Banker et al. (2014) studied the effects of 4 days soft tissue cyclic compressive loads (CCL) of varying forces (0N, 1.4N, 4.5N, and 11N; 30 min) on gene expression and immune response in healthy skeletal muscle. Results indicated that 534 genes were differentially expressed due to massage and 47% of the functional clusters expressed had immunological functions. Gene expression and immune response [Chemokine (C-C motif) receptor-2, Leukocyte immunoglobulin-like receptor (subfamily B, member 4), Cd74 molecule major histocompatibility complex (Class II), Lysozyme 2, and Chemokine (C-X-C motif) receptor-5] varied depending on the load being applied with up- and down-regulation being observed. These findings suggest that immunologic responses induced by massage are likely load dependent in healthy skeletal muscles. Miller et al. looked at the effects of CCL (4.5N, 0.5 Hz, 30 min, every other day for 8 days) on muscle regrowth following atrophy. Significant increases in gastrocnemius cross-sectional area and higher levels of cytosolic and myofibrillar proteins after CCL treatment compared to reloading alone and the contralateral limb (no massage) occurred. Significant increases in DNA synthesis were also observed after CCL compared to reloading (Miller et al., 2018). Additionally, Saitou and colleagues found that local CCL (50 mmHg intramuscular pressure waves, 1 Hz, for 30 min) significantly reduced muscle atrophy (increased cross-sectional area and force production) by modulation of local inflammatory responses (decreased TNF-α-positive, and F4/80- MCP-1-, or TNF-α- double positive) when compared to the contralateral non-massaged hind-limb (Saitou et al., 2018).

Haas and colleagues looked at the effects of varied duration (15 or 30 min), magnitude (5 or 10N) and frequency (0.25 or 0.50 Hz) of massage-like movements on skeletal muscle's recovery after exercise in a rabbit model. After 4 days of massage, significant differences were found for magnitude and frequency with 10N and 0.5 Hz eliciting the best recovery (reduction of myofibrils' damage and leukocyte infiltration), respectively. No statistically significant differences were found for duration (Haas et al., 2013b). This group further investigated the effects of immediate (right after exercise) and delayed (48 h after exercise) massage-like movements (0.5 Hz, 10N, for 15 min) for a total of 4 days on the tibialis anterior muscle recovery. Although significant increases were observed in peak torque output with immediate massage eliciting the greater increases, no statistically significant differences were found for inflammatory cell infiltration (RPN3/57 and CD11b staining) between the two groups (Haas et al., 2013a).

#### Visceral Response

Holst et al. (2005) investigated the effects of 5 min of repeated abdominal stroking (3 or 14 treatments every 2nd day) on plasma levels of gastrointestinal hormones (insulin, gastrin, glucose, and somatostatin) in male rats. Blood samples were collected 10 min after the last massage treatment, plasma separated and radioimmunoassay (gastrin and insulin) and spectrophotometry (glucose) performed. Three sessions of massage significantly decreased plasma levels of insulin and somatostatin, while 14 sessions significantly decreased plasma level of insulin and gastrin, and increased the level of glucose compared to the control group (only handling of the animal). Zhu et al. (2017) determined the effects of low and high intensity (low: 50 g, 50 times/min; high: 100 g, 150 times/min; 14 days) clockwise circular massage and drug administration (intragastric mosapride) on bowel dysfunction in a rodent model of spinal cord injury. Massage therapy showed significant improvement in weight, time to defecation, feces amount, fecal pellet traits, colon histology as well as a significant improvement of interstitial cells of Cajal, c-kit mRNA, and protein levels. Observed improvements were frequent- and pressure-dependent, with high intensity massage eliciting significantly better results compared to low massage intensity and drug administration. Additionally, Chapelle and Bove (2013) investigated the effects of massage (1 min, side to side, and clockwise motions) on gastrointestinal function in a model of post-operative ileus. Overall gastrointestinal function (reduced time to first fecal discharge and improved transit) and reduced intraperitoneal protein and leukocyte levels improved significantly, compared to the non-massaged group.

Bove and Chapelle (2012), Bove et al. (2017) investigated the preventive and treatment effects of abdominal massage (clockwise circles, immediately after surgery or 7 days later) on post-operative abdominal adhesions. Significant reductions in frequency and size of adhesions and a delay in trophic macrophage appearance intraperitoneally were reported. Jay et al. (1986) investigated the effects of digital massage to the closed eyelid (15 min) on intraocular pressure (IOP) and nerve blood flow (ocular and optical nerves). Immediately after massage, both the treated (right) and non-treated (left) eyes showed significant decreases in IOP. Additionally, nerve blood flow was lower during massage and significantly higher after massage for up to 15 min.

#### Gene Expression

Liu and colleagues determined the effects of massage on hepatic gene transfer after intravenous injection of plasmid DNA in mice. Digital pressure was applied using the thumbs to the mice's abdomen in antero-posterior direction using varied repetition and duration regimens. Abdominal massage of four times for 1 s each was the optimal dosage for gene transfer/protein expression in the liver and that this gene transfer is at least partially mediated by pressure and membrane changes in liver cells allowing for simple diffusion of naked DNA into the cell to occur (Liu and Huang, 2002; Liu et al., 2004).

#### Neuroanatomical Effects

Skouras et al. (2009) determined the effects of manual stimulation on axonal collateral branching, muscle reinnervation and functional recovery of vibrissal whisking after nerve reconstruction via facial-facial anastomosis. Five minutes of forward stroking on the whisker delivered daily for 4 months significantly increased whisker movement amplitude and significantly reduced poly-innervated endplates, but showed no statistically significant differences in axonal collateral branching after reconstruction. Raza et al. (2015) investigated the effects of massage-like tactile stimulation (3x daily for 15 min) on neuroanatomy in a rodent model of autism. Significant increases in dendritic branching and spine density in three distinct areas (medial prefrontal cortex, orbital frontal cortex, and amygdala) were observed. Similar neuroanatomical changes and improved motor function were observed after tactile stimulation (3x daily for 15 min) in different models of brain injury (frontal and somatosensory cortex) (Gibb et al., 2010). Tactile stimulation (3 min/day for 33 days) also resulted in reversal of some optic nerve cytoarchitecture changes caused by early iron deficiency (Horiquini-Barbosa et al., 2017).

#### Function and Pathology

Bove et al. (2019) investigated the preventive effects of massage to the forearm on musculoskeletal symptoms and tissue pathology induced by a volitional repetitive task. Massage therapy consisted of 10 cycles of lateral mobilization of forearm flexor muscles, 5 skin rolling over the forearm and long axis stretching of the entire upper limb. Maneuvers were performed for a total of 5 min during 3 weeks of treatment. Massage improved repetitive task performance and decreased discomfort-related rat behaviors. Electrophysiological recordings from the medial nerve showed that the injury + massage group had reduced ongoing neuronal activity in comparison to the injured + non-massage group. Massage also prevented the increase in CD68, neutrophils, collagen deposition and anti-degraded myelin basic protein (DMBP) in the median nerve. Pan et al. (2017) assessed the effects of Tuina massage therapy (0.98N, 30x/minute per acupoint, once daily for 20 days) on motor function, muscle mass and tissue plasminogen activator (tPA) and plasminogen activator inhibitor-1 (PAI-1) levels in a neuropathic pain (sciatic nerve) model. Treatment significantly improved motor function, decreased levels of tPA and PAI-1 and no muscle volume differences were observed. Other massage-related studies investigated its effects on muscle reinnervation/whisking function (Guntinas-Lichius et al., 2007; Grosheva et al., 2008; Ozsoy et al., 2014), nerve injury (Mei et al., 2010), angiogenesis-initiating factors (Ratajczak-Wielgomas et al., 2018), and supraoptic neuroendocrine cells (Myers and Jennings, 1985) with their findings summarized in Supplementary Data Sheet 3.

#### Cellular Response to Simulated *in vitro* Massage

Agarwal et al. (2001) assessed the effects of cyclic tensile strain (CTS) in temporomandibular joint inflammation in an attempt to investigate cellular effects of simulated massage. CTS modulated proinflammatory actions of human recombinant (rHuIL-1b) by suppressing IL-1β, isoform NO synthase (iNOS), COX-2, NO, prostaglandin (PG)E2, matrix metallopeptidase (MMP)-1, and proteoglycan syntheses. Additionally, CTS induced a greater MMP- 2 mRNA expression. Madhavan et al. (2007) found that CTS prevents NF-κB transcriptional activation and induces pro-inflammatory genes by regulating transforming growth factor beta-activated kinase (TAK)1 in its signaling cascade.

### Hybrid Studies

A total of 3 studies were considered as hybrids where the intervention included MT or simulated MT combined with another type of intervention (Pollock et al., 1950; Andrzejewski et al., 2015; Bove et al., 2016). The MT physiological outcomes associated with these combinatorial studies were not interpreted in an isolated manner, thus making it difficult to isolate which physiological outcome was associated with a specific type of therapy. Despite this confounding factor, they still fell within the scope of this review and the decision was made to include them.

Pollock et al. (1950) reported that massage (leg and feet for 5 min) with passive joint mobilization (knee, ankle and toes; 10x per joint) diminished the development of contractures and observed fibrillations but no statistically significant differences in muscle atrophy or histologic appearance were noted. Andrzejewski et al. (2015) assessed the effects of massage (prior and during exercise) on vascular endothelial growth factor (VEGF)-A expression in muscle. Increased VEGF-A expression was observed on both groups (prior and during exercise) after week one indicating that massage likely contributes to the development of new and existing vascular networks in the muscle. Bove et al. (2016) found that massage and joint mobilization decreased deposition of collagen and transforming growth factor beta-1 (TGF-β1) in the forepaw tissues (median nerves and lumbrical muscles) compared to the control group.

## Discussion

The main objective of this scoping review was to identify and summarize physiological changes associated with MT delivered in animal models so as to serve as a resource to better understand and inform future mechanistic and clinical studies involving these increasingly popular integrative approaches to pain management and overall health. While we recognize that MT encompasses many additional forms of therapeutic application, articles for this review were restricted to three commonly used MT clinical interventions (mobilization [*n* = 17], manipulation [*n* = 21], and massage therapy [*n* = 37]). Due in part to the opioid crisis in pain management (Manchikanti et al., 2012; Vadivelu et al., 2018), mechanistic-oriented research interest in non-pharmacological approaches, such as MT, is rapidly growing as evidenced by more than half (53%) of the articles presently reviewed having been published within the last 7 years.

Mobilization interventions were constituted primarily of AJM and KJM studies. Joint mobilization was reported to induce changes in inflammatory profile (Ferretti et al., 2006; Martins et al., 2011; Zhu et al., 2018), gene and protein expression (Ferretti et al., 2006; Wang et al., 2015; Jielile et al., 2016), receptor activation (Skyba et al., 2003; Martins et al., 2012, 2013a,b), neurotransmitter release (da Silva et al., 2015; Santos et al., 2018), oxidative markers and enzymatic activity (Salgado et al., 2019). The majority of mobilization studies supported anti-hyperalgesic/analgesic effects and suggested a variety of potential peripheral and central biological mechanisms were potentially responsible using multiple types of pain models including neuropathic, inflammatory, post-operative, and chronic post-ischemia pain. Reduction of glial hyperactivation and neuropeptide release, coupled with increased endogenous opioid receptor and endocannabinoid system involvement, along with antioxidant enzyme activity collectively point to an increased rationale and need for additional study of joint mobilization in acute and chronic pain management. A present challenge for mobilization, as well as most other MT interventions, is the determination of the optimum treatment frequency and dosage necessary to maximize key physiological changes that sustain diminution of the acute and chronic pain experience. To successfully resolve this optimization of treatment challenge, a stronger scientific emphasis needs to be placed on determining causal mechanisms underlying these aforementioned and other yet to be elucidated physiological responses.

The manipulation studies were unanimously spinal in nature. SM was shown to elicit changes in muscle spindle activity (Pickar and Wheeler, 2001; Sung et al., 2005; Pickar and Kang, 2006; Cao et al., 2013; Reed et al., 2013, 2014a, 2017b), neuronal activity (Song et al., 2006; Reed et al., 2014b,c, 2017a), electromyography (Colloca et al., 2006, 2008, 2012), and immunologic response (Song et al., 2016; Duarte et al., 2019). Manipulation as a therapeutic intervention is distinct from mobilization in that typically a single high velocity, short duration (<150 ms) impulse or manipulative thrust is delivered into a joint. A long-acknowledged mechanistic challenge to clinical manipulation-related research remains the fact that biomechanical characteristics of clinically delivered SM are highly variable and often depend on the specific manipulative technique used, anatomical location to which SM is delivered, the physical complaint, findings on the physical exam or the presence of comorbidities, along with both the clinician's and/or patient's body-type. Evidence presented in this review suggests that peripheral and/or central physiological responses to SM are delivery parameter specific. For example, peripheral muscle spindle afferent response and/or central thalamic neuron activity were shown to be significantly impacted by the manipulative thrust magnitude, thrust duration, tissue preload, anatomic site, and/or joint condition at which the SM was delivered (Cao et al., 2013; Reed et al., 2013, 2014a,b,c, 2015a,b, 2017a,b; Reed and Pickar, 2015). Despite experimentally delivered SM thrusts typically being delivered to a specific vertebra, mechanoreceptor responses to these anatomically well-localized thrusts have been demonstrated to occur several vertebral segments away (Reed and Pickar, 2015) as would be anticipated to occur clinically with even less precise thrust delivery. This demonstration of an SM mechanoreceptor response gradient (Reed and Pickar, 2015) may have clinical implications, not only for SM but for other types of MT as well. The concept of delivery specificity (or clinically a lack thereof) becomes particularly important if it is determined that a specific threshold of MT mechanoreceptor activation is required at a particular anatomical site to elicit a clinical meaningful benefit. There continues to be a great need for mechanistic and clinical studies investigating physiological responses to MT that incorporate different SM dosage delivery parameters. In addition, unlike many of the joint mobilization studies, the majority of reviewed SM studies were performed in animal models which failed to mimic any musculoskeletal pain or pathological conditions. Physiological responses related to SM will no doubt be impacted by tissue inflammation, or neuromusculoskeletal pathology warranting additional preclinical/clinical SM investigations in more relevant models of pain or musculoskeletal pathophysiology.

Massage interventions were applied at various body locations and represented the largest number of published articles out of the three types of MT articles reviewed. Massage therapy was associated with changes in autonomic and circulatory functions (Kurosawa et al., 1995; Lund et al., 1999; Smith and Schober, 2013; Spurgin et al., 2017), lymphatic and immune functions (Wolf et al., 1994; Trubetskoy et al., 1998; Haas et al., 2013a,b; Waters-Banker et al., 2014; Major et al., 2015; Miller et al., 2018; Saitou et al., 2018), visceral response (Jay et al., 1986; Holst et al., 2005; Bove and Chapelle, 2012; Chapelle and Bove, 2013; Bove et al., 2017; Zhu et al., 2017), gene expression (Liu and Huang, 2002; Liu et al., 2004; Jiang et al., 2014), neuroanatomy (Skouras et al., 2009; Gibb et al., 2010; Raza et al., 2015), function and pathology (Vrontou et al., 2013; Pan et al., 2017; Bove et al., 2019), and cellular response to *in vitro* simulated massage (Agarwal et al., 2001; Madhavan et al., 2007; Sowa and Agarwal, 2008). The variability observed in the parameters, applied techniques, body parts, and experimental objectives of massage therapy reflects the broad applicability of this MT intervention and these should be taken into account in future experimental study design. However, such broad application variability can make comparison of physiological effects somewhat difficult, particularly considering that different application parameters (i.e., magnitude) will stimulate different tissues and/or nerve fibers. The overall average of delivery parameters being reported was highest for mobilization studies, followed by manipulation studies and lastly massage studies (Table 2). These five parameters (i.e., force, amplitude, direction, duration, or movement frequency) were selected because they were considered essential to general practice and potentially therapeutic outcomes of MT.

## Limitations

Limitations associated with this scoping review include: (1) selecting publications in English only which could potentially reduce the number of studies being retrieved from the literature search; (2) although not the primary focus of this review, greater analysis of behavioral outcomes associated with these MTs could increase our mechanistic understanding of these therapies; (3) the large variability in delivery parameters (methodology differences even among identified MT techniques), involved tissues, and terminology/definition associated specifically with massage made it more difficult to assess for article inclusion than mobilization or manipulation. Various forms of tactile or soft tissue mechanical stimulation was often termed “massage” by authors, however it was not akin to the type of massage typically delivered for therapeutic purposes in an integrative healthcare setting. Thus, terminology differences used in the field of MT may have resulted in some relevant articles being overlooked or excluded. While this review primarily reports dichotomizations of effects, this strategy allowed a succinct summary of a large and diverse body of literature. A good number of publications failed to report all relevant data (i.e., actual *P*-values, effect sizes), contextual, and/or subordinate factors necessary to make pertinent comparisons within/between MT therapeutic interventions.

## Future Directions

Despite the recent increase in the number of basic science investigations into various physiological changes associated with MT approaches, this review has identified numerous areas that require further study, if indeed the physiological and/or psychological mechanisms underlying MT therapeutic benefits are to be elucidated. Regardless of MT approach, limited evidence exists related to peripheral or central mechanisms involved, and the vast majority of *in vivo* physiological studies typically record outcomes immediately following (or very shortly thereafter) MT delivery. More long-term or longitudinal MT-related preclinical studies are needed as are studies investigating the physiological impact of various MT dosage. Preclinical studies are just beginning to recognize and demonstrate that analgesic modulation related to MT involve complex mechanistic interactions such as endogenous opioid, endocannabinoid, and/or neuroimmune contributions (Vigotsky and Bruhns, 2015), and these effects may require that certain MT dosage thresholds be achieved. Sustained lines of investigation incorporating new and existing preclinical models of neuromuscular pathophysiology and/or somatovisceral pain are needed to investigate each facet of the mechanistic comprehensive model described by Bialosky et al. (2009, 2018). Preclinical studies investigating MT effects on: gene expression, neurotransmitter/neuropeptide/cytokine release, mechanosensitive ion channel activation, neuroimmune response, global cortical/spinal circuit connectivity and descending inhibition, connective tissue stress and strain, neuronal hyperactivity, and synaptic organization are all sorely needed to advance the field and deepen our understanding of MT. As advancements are being made in small animal imaging technology, increased use of these tools will be extremely beneficial in MT preclinical studies allowing more complex and longitudinal study designs with appropriate controls. Use of larger animal models (sheep, pig, etc.) would also prove beneficial allowing MT to be biomechanically applied in a more similar manner to that being delivered in clinical settings.

## Conclusions

Findings from this review suggest that MT approaches elicit numerous and varied physiological changes that alter neural, lymphatic, autonomic, genetic, and molecular responses. Mobilization was shown to modulate nocifensive reflexes potentially via a variety of peripheral and/or central mechanisms, while SM studies clearly demonstrated the importance of delivery parameters to physiological responses occurring at peripheral and/or central levels. Massage therapy was associated with many physiological effects, however due to the wide spectrum of application methodology, terminology, and lack of delivery parameter measurements, collective comparisons and/or relevancy is somewhat difficult. Taken together, these studies highlight the need and importance of capturing and reporting MT delivery parameters as well as the adoption of more uniform operational terminology among MT preclinical and clinical researchers in order to increase rigor, reproducibility and better allow for data comparisons between studies so as to obtain the ultimate goal of improving MT clinical care.

## Data Availability Statement

All data used for analysis and supporting the results of this study are included in this article except for data extraction tables which are available from the corresponding author upon reasonable request.

## Author Contributions

CL contributed to conception and study design, data extraction, data analyses, and manuscript writing. WR contributed to conception and study design, data extraction, data analyses, manuscript writing, and revision. DM contributed to conception and manuscript writing. All authors contributed to manuscript revision, read, and approved the submitted version.

## Conflict of Interest

The authors declare that the research was conducted in the absence of any commercial or financial relationships that could be construed as a potential conflict of interest.

## Abbreviations

AJM: Ankle Joint Mobilization
BW: Body Weight
CAT: Catalase
CCL: Cyclic Compressive Loads
COX-2: Cyclooxygenase-2
CRMP-2: Collapsin Response Mediator Protein 2
CTS: Cyclic Tensile Strain
DRG: Dorsal Root Ganglion
EMG: Electromyographic
EPC: Plain Egg Phosphatidyl Choline
fMRI: Functional Magnetic Resonance Imaging
GABA_A_: γ-aminobutyric acid
HR: Heart Rate
iNOS: Isoform Nitric Oxide Synthase
IL: Interleukin
IOP: Intraocular Pressure
KJM: Knee Joint Mobilization
MMP: Matrix Metalloproteinase
MT: Manual Therapy
MPZ: Myelin Protein Zero
MuRf: Muscle Ring Finger
NGF: Nerve Growth Factor
NO: Nitric Oxide
NS: Nociceptive Specific neurons
PA: Postero-Anterior
PAI-1: Plasminogen Activator Inhibitor-1
PEG-PE: PolyEthyleneGlycol-distearoyl Phosphatidyl Ethanolamine
PKC: Protein Kinase C
SM: Spinal Manipulation
SOT: Superoxide Dismutase
TAK: Transforming Growth factor beta-activated Kinase
TGF-β1: Transforming Growth Factor Beta-1
TNF: Tumor Necrosis Factor
tPA: Tissue Plasminogen Activator
TRPV1: Transient Receptor Potential Vanilloid 1
VGEF: Vascular Endothelial Growth Factor
WDR: Wide Dynamic Range neurons.
