# Supplementary material for: Physiological Responses Induced by Manual Therapy in Animal Models: A Scoping Review
Source: Front Neurosci. 2020 May 8;14:430. doi: 10.3389/fnins.2020.00430 (PMC7227122; doi:10.3389/fnins.2020.00430)
Supplement: Supplementary file 2 [file Data_Sheet_2.docx]

SUPPLEMENTARY FILE B – Search Terms

“Central-mechanism*” OR ((“central nervous system” OR “sympathetic nervous system” OR “autonomic nervous system”) AND mechanism) OR "Neural Pathways" OR “neural pathway” OR "Motor Neurons" OR "Signal Transduction" OR signal-transduction OR signal-pathway OR "Gene Expression" or gene-expression OR "Nervous System" OR "Receptors, Cell Surface" OR neuron* OR “physiological mechanism” OR “physiological response” OR physiology  **AND**

 “spinal manipulation” OR “spinal mobilization” OR massage OR “manual-therap*” OR “manipulative-therap*” OR “manipulation-therap*” OR "musculoskeletal manipulations" OR “musculoskeletal manipulation”  OR “Orthopedic Manipulation” OR “Lumbar Manipulation” OR “Cervical Manipulation” OR “Thoracic Manipulation” OR “Osteopathic Manipulation” OR “Chiropractic Manipulation” OR “Chiropractic Adjustment” OR “Orthopedic Manipulation” OR mobilization-therap* OR “Joint manipulation” OR “joint mobilization” OR “Chiropractic Adjustment”  **AND** “Animal model” OR rat OR rats OR "Rats” OR cat OR cats OR "Cats” OR rodent OR mice OR "Mice” OR sheep OR rabbit OR “rabbits”.
