# Supplementary material for: Physiological Responses Induced by Manual Therapy in Animal Models: A Scoping Review
Source: Front Neurosci. 2020 May 8;14:430. doi: 10.3389/fnins.2020.00430 (PMC7227122; doi:10.3389/fnins.2020.00430)
Supplement: Supplementary file 3 [file Data_Sheet_3.docx]

SUPPLEMENTARY FILE C – DATA SUMMARY TABLES

**1. Mobilization Studies**

| **Studies (Authors, year)** | **Intervention Parameters** | **Specie, N** | **Mechanistic Outcome Measures** | **Main Findings** |
| --- | --- | --- | --- | --- |
| **Ip et al., 2000** | 60˚-135˚; Knee Flexion; 10xDaily for 3 weeks | Rabbits, 14 | Adhesion strength & nerve elongation | No statistically significant differences |
| **Kang et al., 2001** | 25%, 50%, 75%, and 100% BW; 0˚, 45˚ or 90˚; distractive or compressive directions | Cats, 25 | Trunk muscle spindle activity | No statistically significant differences in muscle spindle discharge upon chemosensitization of small diameter afferents |
| **Malisza et al., 2003a** | End of ROM; Knee flexion & Extension; Tibial anterior & posterior translation; 3 x 3min with 1 min rest | Rats, 20 | Cortical Imaging (fMRI) | Capsaicin injection activate brain pain processing areas; No statistically significant differences in activation after knee joint mobilization |
| **Malisza et. al, 2003b** | End of ROM; Knee flexion & Extension; Tibial anterior & posterior translation; 3 x 3min with 1 min rest | Rats, 20 | Spinal cord Imaging (fMRI) | Capsaicin injection activate areas within spinal cord dorsal horn; No statistically significant differences in activation after knee joint mobilization |
| **Skyba et al., 2003** | End of ROM; Knee flexion & Extension; Tibial anterior & posterior translation; 3 x 3min with 1 min rest | Rats, 113 | Neurotransmitter Receptor activation | 5-HT1/2 and α2-adrenergic receptors respectively prevented and reversed joint mobilization antihyperalgesia; No statistically significant differences elicited by GABA_A_ and opioid blockade |
| **Ferretti et al., 2006** | 40°-110°; Knee flexion; 24, 48, 96h or 12 h per day for 2 weeks; 0.022Hz | Rabbits, 25 | Gene activation & IL-10 expression | Repression of pro-inflammatory genes & ↑ IL-10 |
| **Martins et al., 2011** | End of ROM; Ankle dorsi- & plantar flexion; 9 min for 5 weeks | Rats, 48 | Glial activation & Nerve morphological analysis. | ↓CD11b/c and GFAP & ↑ myelin sheath thickness |
| **Martins et al., 2012** | End of ROM; Ankle dorsi- & plantar flexion; 3 or 9 min daily for 6 days | Rats, 32 | Opioid receptor (naloxone) & Leukocyte (fucoidain) migration | Opioid receptors mediate pain ↓; No statistical significant differences for Leukocyte expression |
| **Martins et al., 2013a** | End of ROM; Ankle dorsi- & plantar flexion; 9 min | Mice, 40 | Cannabinoid subtype receptor (CBR) expression | Pain ↓ is mediated by CB_1_R & CB_2_R availability |
| **Martins, et al., 2013b** | End of ROM; Ankle dorsi- & plantar flexion; 10 min | Mice, 32 | Adenosinergic/ Adenosine A1/α-2-adrenergic receptors' & Serotonin expressions | Adenosine A1/α-2-adrenergic receptors' & Serotonin mediate pain ↓ |
| **Ruhlen et al., 2014** | End of ROM; Knee flexion & Extension; Tibial translation (AP); 9 min | Rats, 12 | Gene expression | Inflammatory injury led to differential gene expression; No statistically significant differences observed with mobilization |
| **Wang et al., 2015** | 6%; 9%; 12% elongations; 30°-90°; 30°-130°; 30°-170°; Knee Flexion-Extension; 10xdaily for 4 weeks; 1sec extension-5sec flexion | Rabbits, 30 | Nerve elongation & Gene expression | 9% elongation & ↓ MuRf-1 expression |
| **da Silva et al., 2015** | 30°-45°; Ankle dorsiflexion; 10 sessions of 10 min; 20 oscillations/min | Rats, 24 | Nerve morphology (electron microscopy & Western Blot) | NGF and MPZ mediate nerve regeneration |
| **Jielile et al., 2016** | Squatting and standing; 150±15 times/day | Rabbits, 135 | Protein expression | Early mobilization ↑ CRMP-2, galactokinase 1, tropomyosin-4, and transthyretin expressions |
| **Zhu et al., 2018** | 20°-25° from neutral; Ankle dorsi- & plantar flexion;10 minutes; 5x Weekly for 3 weeks; 20 oscillations/min | Rats, 18 | IL-1β and TNF-α levels | No statistically significant differences between groups; ↓ IL-1β & TNF-α in the nerve trunk+branches between treated/non-treated sides |
| **Santos et al., 2018** | 30°-45°; Ankle dorsiflexion; 10 sessions of 10 min; 20 oscillations/min | Rats, 50 | Neurotransmitter & receptor expression | ↓ Substance P & TRPV1; ↑ µ-opioid receptor |
| **Salgado et al., 2019** | End of ROM; Ankle dorsi- & plantar flexion; 9 min; 40 oscillations/min | Mice, 32 | Oxidative stress (OS), mitochondrial function, protein carbonyls, SOD & CAT levels | Prevention of OS & increase in protein carbonyls and CAT; No statistically significant differences for mitochondrial function & SOD |
| Note: ROM: range of motion; min: minutes; *nr: not reported; IL: interleukin; TNF: tumor necrosis factor; Murf: muscle RING finger; NGF: nerve growth factor; MPZ: myelin protein zero; CRMP: collapsin response mediator protein; TRPV: transient receptor potential vanilloid 1; CAT: catalase; SOD: superoxide dismutase. | | | | |

**2. Manipulation Studies**

| **Studies (Authors, year)** | **Intervention Parameters** | **Specie, N** | **Physiological Measures** | **Main Findings** |
| --- | --- | --- | --- | --- |
| **Pickar & Wheeler, 2001** | 25% and 100% BW; 0˚ & 45˚ to long axis of L6; distraction & compression; From 200ms to 3s (phase dependent) | Cats, 10 | Trunk Golgi Tendon Organ (GTO) and muscle spindle activity | GTO & muscle spindle afferent discharge to impulse phase of SM |
| **Sung et al., 2005** | 33%, 66%, or 100% BW; PA; 25, 50, 100, 200, 400, and 800ms | Cats, 6 | Trunk muscle spindle activity | Afferent discharge ↑ approximately at 100ms; No statistically significant differences for loading profile |
| **Pickar & Kang, 2006** | 33%, 66%, or 100% BW; 25, 50, 100, 200, 400, and 800ms | Cats, 46 | Trunk muscle spindle activity | ↑ primary & secondary muscle spindle discharge as time profile ↓ from 800ms to 50ms. |
| **Song et al., 2006** | Rostrally at 40° to 50° to the vertebral horizontal line; < 0.1ms; Daily for 7 days & every other day on the following week | Rats, 148 | Lumbar DRG neurons & glial cells activities | ↓ excitability (DRG neurons) & Satellitosis (glial cells) |
| **Colloca et al., 2006** | 80N or 20, 40, 60N; PA; 10, 100, 200ms or 100ms | Sheep, 10 | Trunk EMG | No statistically significant differences for variation on pulse durations); variations in force ↑ EMG response |
| **Pickar et al., 2007** | Peak forces 9-30N; 1 or 2mm segmental translation; PA; 12.5, 25, 50, 100, 200, and 400ms | Cats, 54 | Trunk muscle spindle activity | ↑ muscle spindle discharge around 50 to 100ms and at 1mm amplitude. |
| **Wynd et al., 2008** | 20 manipulations | Dogs, 10 | Arterial injury dimension | No statistically significant differences |
| **Colloca et al., 2008** | 80N or 20, 40, and 60N; PA; 10, 100, and 200ms or 100ms | Sheep, 15 | Trunk EMG | ↑ EMG as force ↑; No statistical significant differences in EMG for pulse duration; ↑Compound action potentials for shorter pulse durations; disc degeneration ↑ EMG. |
| **Colloca et al., 2012** | 10% BW (80N); PA; 10 & 100ms; 2, 6 and 12Hz | Sheep, 24 | Trunk EMG | 25-30% ↓ in positive EMG response in the degenerative group; no statistically significant differences in spondylosis group. |
| **Cao et al., 2013** | 25%, 55% or 85% BW; 1, 2 or 3mm thrust displacement; PA; 0-250ms | Cats, 112 | Trunk muscle spindle activity | Resting muscle spindle discharge ↑ at 2mm (all pulse durations), at 2mm (200ms), at 3mm (50ms), 25% BW-25ms and 55% BW-50ms; ↓ at 3mm (75ms) & 85% BW-200ms. No statistically significant differences in ramp or position discharge. |
| **Reed, Long & Pickar, 2013** | 55% BW; PA; 0, 75, 100, 150 & 250ms | Cats, 23 | Trunk muscle spindle activity | Facet fixation: ↑ muscle spindle discharge at 75, 100 & 150ms; Facetoctomy: ↑ discharge at 75 & 100ms. |
| **Reed et al., 2014a** | Preload:18% & 43% of peak applied thrust force; Manipulation: 55% BW; PA; Preload:1 & 4s; Manipulation: 75ms | Cats, 20 | Trunk muscle spindle activity | ↑ muscle spindle discharge during manipulation, ↑ position discharge & ↓ resting discharge upon longer & higher magnitude preload; |
| **Reed et al., 2014b** | 0%, 55% & 85% BW; PA; 100ms | Rats, 15 | Lateral thalamic neurons’ activity | ↑ mechanical threshold in nociceptive specific thalamic neurons at 85% BW compared to 0% in PA direction. |
| **Reed et al., 2014c** | 85% BW; PA; 100 or 400ms | Rats, 9 | Lateral thalamic neurons’ activity | No statistically significant differences in mechanical threshold response of nociceptive specific neurons |
| **Reed et al., 2015a** | Peak forces from 78.2 to 121.8N; PA; 2-3ms | Cat, 1 | Trunk muscle spindle activity | ↑ spindle discharge at preload & thrust; followed by cessation and/or returning to pre-thrust resting levels within 20 or 40s. |
| **Reed et al., 2015b** | Peak force of 21.3N; PA; 100ms | Cats, 16 | Trunk muscle spindle activity | ↑ spindle discharge at all contact sites with higher responsiveness at the targeted vertebra |
| **Reed et al., 2015c** | 55% BW; PA; 0, 75, 100, 150 & 250ms | Cats, 23 | Trunk muscle spindle activity | Lower thrust durations (<150ms) ↑ spindle discharge |
| **Song et al., 2016** | Force setting 1 & 2 on Activator 3 device; Rostrally at 40° to 50° to the vertebral horizontal line; < 0.1ms | Rats, 96 | Neural inflammation, neural excitability and gene expression in the blood, DRG & spinal cord | ↓ DRG neuron hyperexcitability, satellitosis, c-Fos & PKCγ, IL-1β in DRG; ↑ IL-10 in spinal cord. |
| **Reed et al., 2017a** | 22-155N; PA; < 10ms | Cats, 6 | Trunk muscle spindle activity | ↓ spindle discharge & >10s to return to baseline activity |
| **Reed, et al., 2017b** | 85% BW; PA; 100ms | Rats, 54 | Thalamic neurons’ activity | ↓ spontaneous neuronal activity; attenuation of inhibitory evoked response on the contralateral hind-paw |
| **Duarte et al, 2019** | Force setting 1 on Activator 4 device | Rats, 30 | Oxidative markers | Prevention of ↑ in lipid hydroperoxides and NO metabolites; ↓ of catalase activity. |
| Note: BW: body weight; PA: postero-anterior direction; EMG: electromyography; N: Newtons; ms: milliseconds; DRG: dorsal root ganglion; PKC: protein kinase C; IL: interleukin; NO: nitric oxide. | | | | |

**3. Massage Studies**

| **Studies (Authors, year)** | **Intervention Parameters** | **Specie, N** | **Physiological Measures** | **Main Findings** |
| --- | --- | --- | --- | --- |
| **Myers & Jennings, 1985** | 2 min | Sheep, nr* | Supraoptic neuron activity | Non-specific changes in mean firing rate & number of short inter-spike intervals |
| **Jay, Aziz & Green, 1986** | 15 min; 45 sec massage with 15 sec rest | Rabbits, 33 | Blood flow & intraocular pressure (IOP) | Massage ↑ nerve blood flow and ↓ IOP |
| **Wolf et al., 1994** | 9 min | Rabbits, 5 | Nanoparticle accumulation in the lymph node | Massage ↑ transport of subcutaneous nanoparticles to lymph node. |
| **Kurosawa et al., 1995** | 100-150 mmH20; 1 or 5 min; 0.017-0.67Hz | Rats, 28 | Arterial Blood Pressure | ↓ in blood pressure (30-50mmHg) following 1min of massage |
| **Trubetskoy et al., 1998** | 5 min | Rabbits, 17 | Upper body liposome migration (gamma-scintigraphy) | Massage ↑ liposome transport into bloodstream |
| **Lund et al., 1999** | 100 mmH2O; 2 and 5 min; 0.67Hz | Rats, 36 | Blood pressure & Heart rate (HR) | Abdominal massage ↓ blood pressure and HR |
| **Agarwal et al., 2001** | 6% elongation; Equibiaxial strain; 4, 12, 24, or 48h; 0.05Hz | Rabbits, nr* | Gene activation, proteoglycan/NO/PGE2 levels | ↓ IL-1β, iNOS, COX-2, NO, PGE2, MMP-1, and proteoglycan syntheses |
| **Liu & Huang, 2002** | Antero-posterior - abdomen; 1sec each for 4 times | Mice, 35 | Gene expression & Toxicity in the liver | Massage ↑ gene expression in the liver & was non-toxic |
| **Liu et al., 2004** | Antero-posterior - abdomen; 1sec each for 4 times | Mice, 3 | Liver gene expression, receptor activation & Blood pressure | ↑ gene expression; No receptor expression; ↑ blood pressure. |
| **Holst et al., 2005** | 100 mmH2O; 5 min - 3 or 14 times, every other day; 0.67Hz | Rats, 44 | Plasma levels of gastrointestinal hormones | Massage ↓ insulin, gastrin & somatostatin; and ↑ glucose |
| **Guntinas-Lichius et al., 2007** | Forward stroking of right whisker; 5 min/day, 5 days/week | Rats, 112 | Axonal branching (facial nerve); Endplate innervation (levator labii superioris muscle) | No statistical significant differences in branching; ↓ motor endplate poly-innervation |
| **Madhavan et al., 2007** | 12% elongation; 3h; 0.05Hz | Rats, nr* | Gene activation (TMJ) | ↓ TAK1 to inhibit NF-ĸB activation |
| **Groshevaet al., 2008** | Forward stroking of whisker; 5 min/day, 5 days/week for 2 months | Rats, 64 | Axonal branching (facial nerve); Endplate innervation (levator labii superioris muscle) | No statistically significant differences. |
| **Sowa & Agarwal, 2008** | 6% elongation; Uniform circumferential strain; 4h; 0.05Hz | Rats, nr* | Intervertebral disc gene Activation | ↓ matrix metalloprotease-13 & Collagen II; No differences in Collagen I & aggrecan |
| **Skouras et al., 2009** | Forward stroking of right whisker; 5 min/day, 5 days/week for 4 months | Rats, 64 | Axonal branching (facial nerve); Endplate innervation (levator labii superioris muscle) | No statistically significant differences in branching; ↓ motor endplate poly-innervation |
| **Mei, Xu & Li, 2010** | 150±20 Gf; 2mm; 1 min for each point, in total 3 min, 1xday or 5x for each channel, 1xday; 2times/sec | Rats, 144 | Brachial plexus nerve conduction velocity (MNCV); nerve action potential (MNAP); Na+, K+ -ATPase Activity; NGF content | Massage ↑ MNVC, MNAP, Na+, K+ -ATPase Activity and NGF content |
| **Gibb et al., 2010** | Brushing; 15 min, 3xday for 1 week | Rats, 34 | Layer III pyramidal cells tracing | ↑ dendritic arborization in pyramidal cells |
| **Bove et al., 2012** | Medial-lateral and anterior-posterior cecum mobilization; 5 or 10min | Rats, 30 | Visceral Adhesion | ↓ number and severity of adhesion in preventive group (i.e. massage right after surgery) |
| **Chapelle et al., 2013** | Side to side & Clockwise motion; 1 min | Rats, 44 | GI function | ↓ time to 1st fecal discharge & improved transit; ↓ intraperitoneal protein and leukocyte levels |
| **Smith & Schober, 2013** | Rotating motion (nasal) & Digital pressure (eyes); 5-10 sec | Cats, 24 | HR & Doppler velocities | ↓ HR and wave separation |
| **Haas et al., 2013a** | 5 or 10N; 15 or 30 min, for 4 days; 0.25 or 0.5Hz | Rabbits, 24 | Tibialis anterior myofibril damage and cellular infiltration | ↓ myofibril damage & leukocyte infiltration at 10N & 0.5Hz |
| **Haas et al., 2013b** | 10N; Compressive loading; 15min for 4 days; 0.5Hz | Rabbits, 18 | Tibialis anterior cellular infiltration | No statistically significant differences in inflammatory cell infiltration (RPN3/57 and CD11b staining) |
| **Vrontou et al., 2013** | Brushing of intact hairy skin; Several trials of 1 or more stimuli separated by few min | Mice, 2 | Calcium imaging of MrgprB4+ neurons | Activation of MrgprB4+ neurons upon brushing stimulus, but not upon pinching stimulus |
| **Waters-Banker et al., 2014** | 0, 1.4, 4.5 and 11N; Compressive loading; 30min, once a day for 4 days; 0.5Hz | Rats, 24 | Tibialis anterior gene expression & Immune cell modulation | Varied & load dependent up- and down-regulation |
| **Jiang et al., 2014** | Pinch (back); 20 times/day for 1 week | Rats, 50 | Hippocampus gene expression | ↓ differential gene expression |
| **Ozsoy et al., 2014** | Forward stroking of whisker; 5 min/day, 5 days/week for 4 months | Rats, 48 | Axonal branching (facial nerve); Endplate innervation (levator labii superioris muscle) | No statistically significant differences |
| **Major et al., 2015** | 100-150 mmH2O; Brush or hand stroking from thoracic to superior hind-limb region; 60 min, for 8 days; 0.5-1Hz | Mice, 18 | Cellular quantification (thymus & spleen) and noradrenergic innervation of lymphoid organs | ↑ number of thymocytes, CD4+ CD8+, CD4+ and CD8+ subpopulations; ↓ noradrenergic innervation of lymphoid organs |
| **Raza et al., 2015** | 3 times/day for 15 min | Rats, 12 | Dendritic architecture of layer III pyramidal cells of the medial & orbital prefrontal cortices, and [amygdala](https://www-sciencedirect-com.ezproxy3.lhl.uab.edu/topics/neuroscience/amygdala) | ↑ dendritic branching and spine density in different brain areas |
| **Horiquini-Barbosa et al., 2017** | Cephalocaudal (neck to lower back); 3 min once a day for 33 days | Rats, 72 | Optic nerve cytoarchitecture | Massage reversed oligodendrocyte, damaged fiber and myelinated fiber density; No statistically significant differences in astrocyte density |
| **Pan et al., 2017** | 0.98N; Pressing, strumming and circular rubbing (GB30, GB34 and BL57 acupoints) ;3 min, once a day for 20 days; 30 times/min | Rats, 48 | Gastrocnemius muscle volume & tPA and PAI-1 levels | No statistically significant differences in muscle volume & ↓tPA and PAI-1 levels |
| **Spurgin et al., 2017** | 20mmHg or 40mmHg; stroking (abdomen); 5 min | Rats, 13 | Blood pressure | ↓ blood pressure |
| **Zhu et al., 2017** | 50g or 100g; 5mm; clockwise circles; 3 min/side for 14 days; 50 times/min or 150 times/min | Rats, 64 | Intestinal function; Gene & Protein expressions; Tissue morphology. | ↑ Intestinal function; ↑ c-kit mRNA and protein levels; ↑ number, distribution, and ultrastructure of colonic interstitial cells of Cajal. |
| **Bove et al., 2017** | Clockwise circles; 1 min every 4h, for a maximum of 4 days | Rats, 147 | Primary adhesion area; Intraperitoneal adhesion severity; Intestinal function; Macrophage phenotype. | ↓ cohesive adhesion; delayed M2 migration intraperitoneally. |
| **Miller et al., 2018** | 4.5N; Cyclic compressive loads; 30 min, every other day for 8 days; 0.5Hz | Rats, 32 | Gastrocnemius muscle morphology & gene expression | ↑ cross sectional area, protein levels & DNA synthesis. |
| **Ratajczak-Wielgomas et al., 2018** | Spiral movements; 5 min, 5x/week for 7 weeks | Rats, 48 | Gene expression of proangiogenic factors | ↑ Vascular endothelial growth factor (VEGF)-A, FGF-2 and CD34 |
| **Saitou et al., 2018** | 50 mmHg; Cyclical compression; 30 min; 1Hz | Mice, 24 | Triceps surae morphology & inflammation | ↑ cross-sectional area & force production; ↓ TNF-α-positive & F4/80- MCP-1- or TNF-α- double positive |
| **Bove et al., 2019** | Lateral mobilization; skin rolling; and long axis stretching of the entire upper limb; 5 min for 3 weeks | Rats, 34 | Neuronal activity (C & A-delta fibers), median nerve injury & inflammation | ↓ ongoing neuronal activity and conduction velocity; Prevention of CD68, neutrophils, collagen deposition and DMBP in the median nerve |

Note: min: minutes; sec: seconds; *nr= not reported; ROM: range of motion; TMJ: temporomandibular joint; N: Newtons; Hz: Hertz; Gf: grams-force; IL: interleukin; iNOS: isoform nitric oxide synthase; COX-2: cyclooxygenase-2; NO: nitric oxide; PGE2: prostaglandin E2; MMP: matrix metalloproteinase; TAK1: transforming growth factor beta-activated kinase-1; NF-ĸB: nuclear factor kappa B; Na: sodium; K: potassium; tPA: tissue plasminogen activator; PAI-1: plasminogen activator inhibitor-1; DNA: deoxyribonucleic acid; TNF: tumor necrosis factor; DMBP: anti-degraded myelin basic protein.

**4. Hybrid Studies**

| **Studies (Authors, year)** | **Intervention parameters** | **Specie, N** | **Physiological Measures** | **Main Findings** |
| --- | --- | --- | --- | --- |
| **Pollock, et al., 1950** | Full ROM; knee flexion/extension & ankle dorsi-/plantar flexion; 10 x/ea. joint; 5 minutes, 36 to 50 stroking & 25-35 kneading | Cats, 68 | Muscle atrophy & fibrillation (leg); Nerve regeneration (sciatic nerve) | No statistically significant differences |
| **Andrzejewski et al., 2015** | Spiral movements; 5 minutes, 5x/week for 3 weeks | Rats, 75 | Vascular endothelial growth factor (VEGF)-A expression in flexor digitorum brevis muscle | ↑ VEGF-A |
| **Bove et al., 2016** | Traction, PA, axial movements, back-and-forth rolling of the forearm tissues; 5 days/week for 12 weeks | Rats, 28 | Median nerve collagen and TGF-β1 deposition | ↓ Collagen and TGF-β1 tissue deposition |

Note: ROM: range of motion; PA: postero-anterior; TGF-β1: transforming growth factor beta-1
